# Unraveling honest responding: a systematic review on the effectiveness of social desirability bias reduction methods in survey research

**DOI:** 10.1007/s11135-026-02664-7

**Published:** 2026-03-14

**Authors:** Emma Zaal, Yfke Ongena, Nina van der Velden, Dan Loughnan, John Hoeks

**Affiliations:** 1 Faculty of Arts, University of Groningen, Groningen, The Netherlands; 2 Faculty of Social Sciences, Radboud University, Nijmegen, The Netherlands

**Keywords:** Systematic review, Social Desirability Bias (SDB), Survey methodology, Face-saving, List experiment, RRT/NRRT

## Abstract

Social Desirability Bias (SDB), the tendency of respondents to present themselves in socially acceptable terms, poses serious challenges for the validity of survey research. This study systematically reviewed the effectiveness of methods aimed at reducing SDB. Searches in Scopus and PsycINFO of publications from 2017 to 2021 identified a total of 121 experiments in 79 peer-reviewed papers. These experiments, conducted in over 20 Western countries, employed 13 SDB-reduction methods across more than 17 behavioral or cognitive topics. Based on 10 quality measures, the methodological quality of these experiments was typically high. There was considerable variability in the frequency of SDB-reduction methods being used and their effectiveness. The most common methods were list experiments, probability-based techniques (RRT/NRRT), face-saving strategies, survey mode, and proxy reporting. Overall, in 55% of experiments SDB was significantly reduced, with face-saving strategies demonstrating the highest efficacy. Future research should aim at replicating these findings in a wider range of (societally relevant) topics and more comprehensively testing promising less explored methods. This review highlights the importance of continuing to refine and test SDB reduction techniques to improve survey data quality.

**Supplementary Information:**

The online version contains supplementary material available at 10.1007/s11135-026-02664-7.

## Introduction

Socially desirable responding, or Social Desirability Bias (SDB), refers to the tendency of individuals to provide inaccurate self-reports in assessments, surveys and interviews in order to present a favorable image of themselves (van de Mortel 2008). SDB can take the form of impression management and self-deception (e.g., Perinelli and Gremigni 2016; Holtgraves 2004). Impression management, by which one consciously deceives, occurs due to a need for social approval. Self-deception, by which one believes one’s own inaccurate self-reports, is a more unconscious way in which we hide our true selves and arises from a need to maintain a positive self-image (Krumpal 2013). Both impression management and self-deception are inevitable in daily communication, and can manifest in any type of social situation. SDB is likely when a given topic is associated with evident social norms. The extent to which our self-disclosures deviate from the truth also depends on the topic under discussion and its perceived sensitivity in the communicative context. In addition, it depends on whom we are talking to (Bäckström and Björklund 2014). For instance, students’ overreporting of alcohol consumption could be regarded as SDB towards fellow students, while underreporting alcohol consumption might be regarded as SDB towards their parents.

Research situations involving questionnaires and interviews are essentially social interactions, and as such, they are sensitive to SDB. SDB contaminates self-report data causing individuals to provide answers to questions that deviate from true values and by increasing the likelihood of item non-response (leaving questions unanswered) (Tourangeau and Yan 2007). Results of subsequent analyses then become biased (e.g., Jann et al. 2019; Kwak et al. 2019). Still, surveys and interviews are important tools in measuring human behavior, cognitions (e.g., intentions, attitudes) and personality characteristics. We rely heavily on self-report measures to develop theory, construct scientific models, and build evidence-based interventions aimed at behavior change (e.g., Noar et al. 2018; Fishbein and Ajzen 2010; Zaal et al. 2023). Ideally, we are able to collect and analyze accurate, unbiased data from self-reports. However, when answering questions, respondents always make choices on their self-presentation and what they report. It is generally recognized that SDB is a serious concern that needs unraveling of its underlying mechanisms and complex interplay of personal and situational characteristics. Personal characteristics include respondents’ age, gender and personality (Chung and Monroe 2003). Situational characteristics include item topics (e.g., healthy or sustainable behavior, Verhoef and van Doorn 2016); item formulation (e.g., formulating survey questions most optimally in terms of comprehension and accurate answering, see Krumpal 2013); and context features (e.g., mode of administration, see Holbrook et al. 2003). While a variety of methods aimed at reducing SDB are available and a larger body of research is developing, what the most optimal methods are to reduce SDB remains elusive (Horiuchi et al. 2019; Erdmann 2019; Wolter 2019; Andersen and Mayerl 2019; Franzen and Mader 2019; Krumpal 2013; Haan et al. 2017).

Hence, above all, there is a need to learn more about effectively reducing SDB. A crucial step for a deeper understanding of SDB reduction methods is to review the literature within this growing research area. A variety of reviews, systematic reviews and meta-analyses on SDB have been published in the last decade (e.g., Krumpal 2013; Dodou and Winter 2014; Perinelli and Gremigni 2016; Gnambs and Kaspar 2017; Cerri et al. 2019; Vesely and Klöckner 2020; Lanz et al. 2021). However, none of them systematically addresses recently published experimental studies that include a diversity of methods aimed at reducing SDB. To this end, our systematic review (cf.: Carvalho et al. 2019) provides an overview and explanation of recently investigated SDB reduction methods and highlights those that synthesis show to be most effective. We include in our review only experimental studies (i.e., correlational studies are excluded). This review outlines experiment’s characteristics (e.g., behavioral/cognitive topics, sample information, example operationalizations), evaluates experimental quality, and provides future directives for SDB reduction research.

## Methodology

### Protocol and registration

The protocol of this systematic review was registered, and accessible through PROSPERO (registration code CRD42022314350). We followed PRISMA (Preferred Reporting Items for Systematic Reviews and Meta-Analysis) guidelines, an evidence-based protocol for synthesizing reporting in systematic reviews and meta-analyses (Moher et al. 2010).

### Eligibility criteria

Formulating inclusion criteria, we followed the PICOTS mnemonic, including a description of the population, the intervention, a comparator, a primary outcome of interest, time and study design. PICOTS facilitates the process of clearly defining the criteria for systematic reviews that are focused on intervention-based research (Carvalho et al. 2019). We formulated three other criteria for inclusion. First, the article must have been published in a peer-reviewed journal. Second, the article must have been written and published in English, to make the review verifiable and replicable for a broad academic community. Third, we excluded topics such as job interviews and performance reviews, as these behavioral topics pertain to contexts that inherently involve subjects presenting themselves in the most socially desirable light. Table 1 summarizes our inclusion criteria based on PICOTS. The rationale for the inclusion criteria are as follows:

- *Population* We aimed to include heterogeneous populations (i.e., with various subpopulations) while maintaining sufficient homogeneity to draw solid conclusions. Although rapidly evolving (e.g., Rosenzweig et al. 2020), survey research remains less developed in non-Western countries. Research institutes adhering to established extensive protocols are predominantly Western (e.g., AAPOR 2024; ESRA 2024). Therefore, assuming a larger degree of homogeneity across Western cultures compared to non-Western cultures (cf. Morris et al. 2015; Lalwani et al. 2006) we included studies that investigated SDB within Western populations.
- *Intervention* interventions manipulating topic, context, question wording and questionnaire procedure characteristics aimed at reducing SDB (i.e., experimental conditions) were included. Examples include interventions manipulating question and answer option formulation/framing or levels of anonymity (Krumpal 2013; Tourangeau et al. 2000). Interventions that measured behavior, cognitions (e.g., attitudes, intentions) and/or personality characteristics (e.g., extraversion, moral self-image) were included.
- *Comparator* comparator interventions were considered conditions to which the SDB-manipulation was compared to (control conditions, or other experimental conditions).
- *Outcome* the extent to which SDB was reduced, determined by inspecting significant differences in answering behavior between conditions (*p*-level set at 0.05).
- *Time* studies published from 2017 to 2021.
- *Study design* Experimental designs with at least one manipulation aimed at reducing (or inducing) SDB were eligible for inclusion. Between- and within-subject experimental designs were included. Correlational studies on SDB were excluded, such as studies investigating the relation of scores on an SDB scale and behavior or individual characteristics like gender, level of education and cognitive measures (e.g., Perinelli and Gremigni 2016).

**Table 1:** Inclusion criteria based on PICOTS mnemonic

PICOTS	Inclusion criteria
Population	A (sample of a) population of Western adults (Including Europe, United States, Australia and New Zealand)
Intervention	Experimental condition including manipulation aimed at reducing SDB (i.e., experimental condition)
Comparator	Control or other experimental condition without SDB manipulation.
Outcome of interest	Extent of SDB reduction
Time	Publications from 2017 to 2021
Study Design	Experimental

### Information sources and search strategy

Based on Gusenbauer and Haddaway (2020), who extensively compared the systematic search qualities of 28 of the most widely used academic search systems, we decided to consult two academic databases: PsycINFO and Scopus. We chose PsycINFO because it focuses on research carried out in the fields of psychology, social sciences and behavioral sciences, in which SDB is a frequently studied phenomenon. Still, studies on SDB are not limited to these disciplines, and therefore we also included Scopus, which is the largest peer-reviewed multidisciplinary database. We decided to not carry out (additional) searches through Google scholar (e.g., like the systematic reviews on SDB of Perinelli and Gremigni 2016 and Dodou and Winter 2014), as Google Scholar is not Boolean functional and the results of Google Scholar searches are dependent on individual searches and are as such, not replicable (Gusenbauer and Haddaway 2020). For Scopus, we used the following Boolean syntax to search within abstracts (= ABS) only: ABS (“social* desirab*” OR “respon* bias” OR “faking”) AND PUBYEAR > 2017 AND PUBYEAR < 2022. For PsycINFO, we used the same search terms and manually selected “search within abstract”, and publication date “between 2017–2021”. In order to ensure an appropriate search balancing the sensitivity and specificity of the search, the full search strategy was inspected and approved by an information specialist of the University of Groningen.

### Study selection

**Fig. 1:**
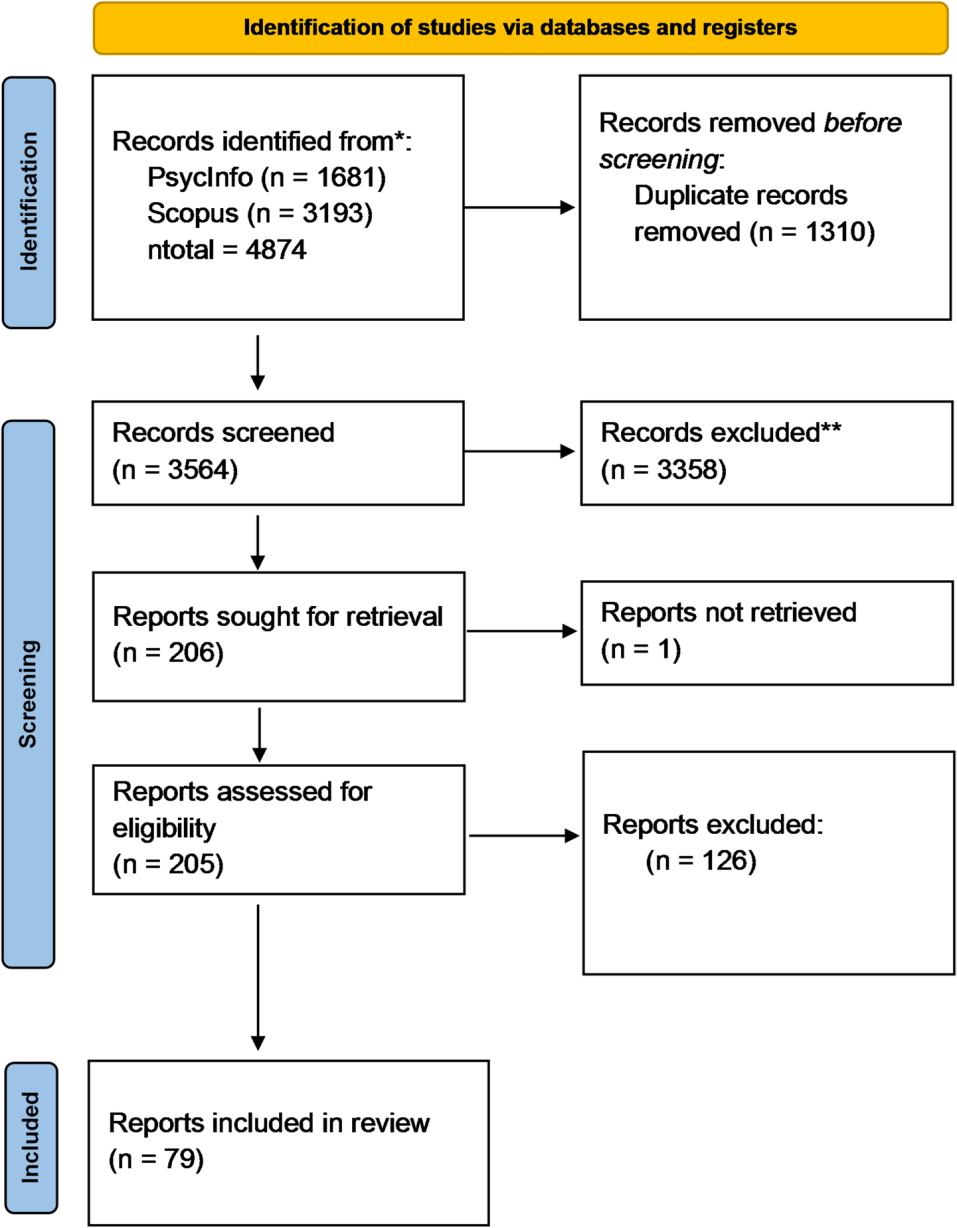
PRISMA flow diagram

Two reviewers (EZ and DL) independently screened 3564 abstracts for inclusion or exclusion in the systematic review with screening software Rayyan (rayyan.ai). Differences in decisions between the screeners were resolved by discussion. If necessary, a third independent reviewer was consulted (JH or YO). 205 Full texts were screened by the same reviewers (EZ and DL). Again, disparities were resolved by discussion. Figure 1 shows the PRISMA flow diagram (Page et al. 2021) of the abstract and full text screening process, including the number of reports included in the review.

### Data collection process and data items

We used Cochrane guidelines (Higgins et al. 2022) to identify required reporting criteria for conducting a systematic review. We supplemented these criteria with specific requirements tailored to the objectives of our review, integrating them into a data extraction sheet. This sheet served as a tool for gathering study information and conducting quality assessments. Table 2 outlines all data items extracted from each experimental study.

The data items shown in bold in Table 2 were double-extracted (independently coded by two reviewers EZ and NV) for experiments reported in just under half of the papers (*n* = 35). Disagreements in data extracted were settled through dialogue between these reviewers. Again, whenever consensus could not be reached, a third independent reviewer (JH or YO) was consulted. After double extraction of 30% of all experiments, saturation in coding was reached (i.e., no disagreements were encountered during data extraction anymore). Data extraction for the remaining 70% of the experiments was done by one reviewer (EZ).

**Table 2:** Data item extraction list

Data items	Data abstracted
Details Citation	Authors, title, publication year, journal of publication, doi.
Topic(s) of self-report	General topic (e.g., health) and specific topic (e.g., alcohol consumption).
Theoretical framework, objective and expectations	Method/theoretical framework used in the study; objective/goal of study.
Sample	**N participants; country; age; gender**; **sampling frame** (i.e., from which population was the sample drawn); **sample recruitment** (e.g., probability/non-probability); **incentives given** (Yes/No/Unclear/Mixed)
Methods	**Design** (number of groups/comparisons); **mode of administration/study context** (e.g., self-administered online survey, laboratory setting); **main independent/dependent variables & operationalization; covariates.**
Outcomes	**Statistical analyses carried out; primary outcomes; effect sizes mentioned** (Yes/No); **Primary outcome** (e.g., experimental condition(s) reduced, increased or had no effect on SDB); **implications for questionnaire design** (i.e., method supported, mixed/unclear, unsupported).

### Quality assessments

In order to assess the quality of the experiments that were included in this review, we used an adapted version of the Downs and Black Checklist (Downs and Black 1998). Table 3 shows the quality items and the criteria that were used for quality assessment.

### Synthesis of results

The interventions and outcome measures of the studies included in this systematic review differed largely and a quantitative analysis or meta-analysis of results was perceived inappropriate. We synthesized our findings and present our data in accordance with the Synthesis Without Meta-analysis (SWiM) guideline (Campbell et al. 2020), where possible and relevant (i.e., as SWiM guideline primarily caters to healthcare interventions, not all of its recommendations were directly applicable to our review).

**Table 3:** Assessment of study quality checklist

Quality items	Study quality characteristic	Description
Reporting	Characteristics of participants clearly described	Were the participants characteristics for the full sample or the separate conditions clearly described in terms of gender and age? (1 = yes, 0.5 = intermediate, 0 = no)
	Interventions clearly described	Is the intervention (i.e., the method used to reduce SDB) clearly described in the paper? (1 = yes, 0.5 = intermediate, 0 = no)
	Main findings clearly described	Are the main findings clearly described? Is it clear whether an effect was found for the method employed to reduce SDB? (1 = yes, intermediate = 0.5, 0 = no)
External validity	Participants representative	Were the subjects who participated representative for the entire population from which they were recruited? If quota sampling, are participants representative at least for gender, age and one other characteristic (such as political preference, education level, income etc.)? (1 = yes, 0.5 = intermediate, 0 = no)
Internal validity – investigator bias	Statistical tests appropriate	Are the statistical tests that were carried out appropriate? (1 = yes, 0.5 = intermediate, 0 = no)
	Outliers	Are outliers mentioned (if applicable based on data type)? (1 = yes / n.a., 0 = no)
Internal validity – selection bias	Even/proper distribution participants	Are participants evenly (with 10% divergence between conditions maximum) or properly (i.e., divergence necessary for statistical power) distributed over conditions? (1 = yes, 0 = no/unclear). If not even, state N per condition.
	Random distribution participants	Are participants randomly distributed between conditions? (1 = yes / n.a. 0 = no / unclear)
Construct and measurement validity/reliability	Measurements	Are measurements of sensitive questions reliable and valid? (1 = yes, 0.5 = intermediate, 0 = no)
	Manipulations	Are manipulations of the method aimed at lowering SDB reliable and valid? (1 = yes, 0.5 = intermediate, 0 = no)

## Results

### Study information

In the 79 reports included in this review (see Fig. 1), 121 experiments were reported. These experiments took place in 23 countries, with 2 experiments not specified on a country level. Most experiments were carried out in the United States, followed by Germany, together making up for almost two-thirds of the experiments included in this review (for an overview of all countries see supplemental material). Most studies employed a between-subject design (*N* = 104; 86%), with the number of conditions ranging from 2 to 15. A small number of experiments had a within-subject design (*N* = 12; 10%) or had a mixed design, containing within-, and between-subject design elements (*N* = 5; 4%). Experiments in which mode of administration was *not* manipulated as SDB reduction method (*N* = 105; 87% of all experiments), used a total of 109 modes, with the majority being self-administered surveys (*N* = 93), followed by interviews over the phone (*N* = 8) and face-to-face interviews (*N* = 7). For one experiment, it was unclear which mode of administration was used.

### Sample type and size

The 121 experiments used 124 different samples, as shown by Table 4 (i.e., three experiments used two different sample types in creating one sample). The majority (*N* = 103; 83%) used non-probability samples, with convenience sampling being the most common (*N* = 52; 50%) of all non-probability samples, followed by quota sampling (*N* = 38; 37%). For the 21 (17%) probability samples, the most common sample was a cluster and/or stratified sample (*N* = 16; 76%), followed by random digit dialing (*N* = 3; 14%). The cumulative sample size was 182.476. Four experiments did not specify sample size. Most studies had a sample size between 500 and 999 (*N* = 25; 21%) or 1000–1999 participants (*N* = 33; 27%), as indicated by Table 4.

**Table 4:** Types of samples and sample sizes of experiments

Type of sample	*N* total (%)
*Probability total*	21 (100)
Cluster/stratified	16 (76)
Random Digit Dialing	3 (14)
Unspecified	2 (10)
*Non-probability total*	103 (100)
Convenience	52 (50)
Quota	38 (37)
Purposive	4 (4)
Snowball	1 (1)
Unspecified	8 (8)
*Sample size*	
0–249	21 (17)
250–499	13 (11)
500–999	25 (21)
1000–1999	33 (27)
2000–3499	12 (10)
> 3500	13 (11)
?	4 (3)
Total	121 (100)

### SDB reduction methods

We identified 13 distinct types of SDB reduction methods across the 121 experiments reviewed. Tables 5, 6, 7, 8, 9, 10, 11, 12, 13, 14, 15, 16, 17 presents all the method types included in this systematic review, along with descriptions of each, example operationalizations, an outline of important advantages and disadvantages of each method and list references to all papers that used the corresponding method.

**Table 5:** List experiment description, example operationalization, advantages, disadvantages and papers of publication

Description	Example operationalization	Advantages and disadvantages
In a list experiment (or Item-Count-Technique), participants indicate for a list of statements how many they agree with, without specifying exactly which ones they agree with. In a typical case, the control group sees 4 statements that are not of interest, while the experimental condition sees the same 4 statements plus 1 sensitive statement of interest. Differences between conditions reveal potential SDB. Attributed to increased anonymity, list experiments are expected to lead to less SDB (e.g., Beiser-Mcgrath and Bernauer 2021).	Example from Beiser-McGrath and Bernauer 2021, p.4–5) Participants were asked: How many of these statements do you believe in? Control group (4 statements): 1. Raising the minimum wage to 12 euros would put many companies out of business. 2. Adding a maximum speed limit on the Autobahn would reduce traffic fatalities 3. Free trade agreements, such as TTIP, would worsen product and food standards in Germany 4. Reducing the use of nuclear power would cause CO2 to increase and worsen the environment. Treatment (experimental) group: 1–4 = identical to control group + the sensitive item: 5. Global warming/climate change is not caused by humans	**Advantages**: + Enhances perceived anonymity, as participant can avoid direct answers + Applicable across behavioral domains + Well-established in a substantial body of evidence **Disadvantages**: - Vulnerable to design effects (response to one item can influence others) - Relies on truthful responses to control items - Requires large sample sizes - Only provides aggregate-level results - Involves complex statistical analysis

Agerberg, M. (2020)*, Beiser-McGrath and Bernauer (2021)*, Blom-Hansen et al. (2021)*, Bromberg et al. (2018)*, Brownback and Novotny (2018)*, Carmines and Nassar (2021), Creighton et al. (2019)*, Creighton and Wozniak (2019), Gaia and Al Beghal (2019), Kleykamp et al. (2018)*, Krumpal et al. (2018), Lai et al. (2021)*, Mishel (2020)*, Morning et al. (2019)*, Mueller (2021)*, Munzert and Selb (2020), Rinken et al. (2021)*, Tan et al. (2021), Thomas et al. (2017), Timmons et al. (2021)*

*Significantly reduced SDB by using list experiment

**Table 6:** Probability-based techniques descriptions, example operationalizations, advantages, disadvantages and papers of publication

Description	Example operationalization	Advantages and disadvantages
Probability-based techniques are rooted in probability theory. They consist of so-called randomized response techniques (RRTs) and non-randomized response techniques (NRRTs). In a RRT, participants are instructed to utilize a randomizing procedure or device (i.e., roll a dice or pick a card). Participants know the outcome of the procedure or device, and based on this outcome they have to answer questions. It is assumed that participants expect that the researcher does not know this outcome and that due to increased anonymity, SDB is reduced—while in fact, the outcome can be calculated on the basis of probability theory. There are several variants and non-randomized adaptations of the RRT (e.g., Erdmann 2019.)	Example Randomized Response Technique (RRT) from Cobo et al. (2021, p.5–6) Participants were asked: Have you ever consumed illegal drugs? Control group: Answer options: Yes / No Experimental group: Instruction for answering: 1. Select a card. 2. If the card chosen is number 1 or 2, your answer to the question must be “Yes”, regardless of the true answer. If it is number 3 or 4, your answer must be “No”. If it is 5, 6 or 7 or a figure, please answer the question honestly. 3. Do not tell the interviewer which card you have chosen (to maintain your anonymity about the answers given). 4. Return the card to the deck and repeat the process for the other questions.) Example Non-Randomized Response Technique (NRRT): triangular model (Erdmann 2019, p. 149–150) Did you ever use prescriptive medication for enhancing mental performance? Control condition Answer options: Yes / No Experimental condition: Additional question: Is your mother’s birthday in January, February, or March? Answer options: The answer is “no” on both questions / The answer is “yes” on at least one of the questions	**Advantages**: + Enhances perceived anonymity, as participant can avoid direct answers + Applicable across behavioral domains **Disadvantages**: - Higher estimates don’t guarantee validity (“more-is-better” fallacy) - Provides only aggregate-level estimates, not individual-level data - Large sample sizes required - Prone to non-compliance; misunderstanding or ignoring the randomizer (especially RRT) - High cognitive burden; instructions and tools can confuse or disengage respondents (especially RRT) - Estimates may not align across studies, even with similar designs and populations (especially RRT) - Most common study design of NRRT is not well-suited for validation, as it cannot separate reduced SDB from random responses or non-compliance

Cobo et al. (2021)*, Höglinger and Jann (2018), John et al. (2018)

*Significantly reduced SDB by using RRT

Canan et al. (2021)**, Erdmann (2019), Hoffmann et al. (2020), Hoffmann and Musch (2019)**, Höglinger and Jann (2018), Meisters et al. (2020a)**, Meisters et al. (2020b)**, Mieth et al. (2021)**, Waubert de Puiseau et al. (2017)**, Wlömert et al. (2019)**

**Significantly reduced SDB by using NRRT

**Table 7:** Face-saving strategies, description, example operationalization, advantages, disadvantages and papers of publication

Description	Example operationalization	Advantages and disadvantages
Face-saving strategies or forgiving wording consist of adapting conventional question and answer option formulation in order to loosen conventional social norms. One or both of the following manipulations are utilized: (1) adding a face-saving preamble before asking the question, and; (2) offering face-saving answer options. It is assumed that the face-saving strategy softens social norms, and as such, leads respondents to be more willing to admit to norm-noncompliant behavior (Daoust et al. 2021a, b)	Example from Daoust et al. ( 2021b, p.4) [Please note that this experiment was carried out during a COVID-19 lockdown] Participants were asked: Have you done any of the following activities in the last week? Go shopping […]; Meet friends, family [.]; Have a group […]; Participate in social […]. Control group: Yes / No Experimental group: Face-saving preamble: “Some people have altered their behaviour since the beginning of the pandemic, while others have continued to pursue various activities. Some may also want to change their behavior, but cannot do so for different reasons.” Guilt-free answer options in italic: Yes / Occasionally / Only when necessary / No	**Advantages**: + Applicable across behavioral domains + Relatively easy to design and implement *preambles and forgiving question wording* **Disadvantages**: - Potentially difficult to craft face-saving *answer options*—especially when designing face-saving alternatives for “no” - Unclear whether preamble and/or answer options are more effective in reducing SDB

Charles and Dattalo (2018)*, Daoust et al. (2021a)*, Daoust et al. (2021b)*, Setzler (2018)*

*Significantly reduced SDB by using face-saving strategy

**Table 8:** Modes of administration description, example operationalization, advantages, disadvantages and papers of publication

Description	Example operationalization	Advantages and disadvantages
In general, it is assumed that a self-administered web survey is the least prone to SDB compared to other modes (especially interviewer-administered mode, cf. Berzelak and Vehovar 2018) due to increased anonymity (e.g., Abrajano and Alvarez 2019).	Example of Berzelak and Vehovar (2018, p.24) Mode: web Self-administered online questionnaire Mode: computer-assisted personal interviewing (CAPI) In person, interviewer-administered oral interview. Computer used by the interviewer. Mode: Computer-assisted telephone interviewing (CATI) Remote, interviewer-administered oral interview. Computer used by the interviewer.	Advantages web surveys**: + Cost-effective for large-scale data collection + Easy to automate, distribute, and standardize across diverse samples Disadvantages web surveys**: - Higher non-response rates relative to other survey modes - Increased risk of respondent fatigue, particularly in longer surveys - Prone to satisficing (i.e., reducing cognitive effort by selecting an adequate rather than optimal answer) - Limited access to hard-to-reach or digitally excluded populations

Abrajano and Alvarez (2019)*, Berzelak and Vehovar (2018)*, Cea D’Ancona (2017)*, Cernat and Sakshaug (2020), Fail et al. (2021), Gamblin et al. (2017), Kisala et al. (2019), Klein et al. (2020)*, Knox et al. (2020)*, Liu (2017)*, Matel and Poskrobko (2019), Meixner et al. (2020), Schuetzler et al. (2018), Stark et al. (2019), Triga and Manavopolous (2019)*, Zhang et al. (2017)*

*Significantly reduced SDB by manipulating mode of administration

** Given that this review primarily examines methods used in online survey contexts, we specifically highlight the advantages and disadvantages of web-based survey administration

**Table 9:** Proxy reporting description, example operationalization, advantages, disadvantages and papers of publication

Description	Example operationalization	Advantages and disadvantages
Proxy reporting is the practice of obtaining information from someone about the behavior or cognitions of another individual or group. This approach operates under the premise that topics become less sensitive when one is able to externalize potentially unfavorable behavior and psychological states onto other people. As such, one is less prone to SDB when answering for someone else compared to answering for yourself (e.g., Kilian and Mann 2021).	Example from Kilian and Mann (2021, p.5) Participants were asked to what extent they agreed with several statements on a Likert-scale. Control group, direct questioning: “How much do you agree with the following statements?” Experimental group, indirect questioning (proxy) condition: “How much do you think ‘a typical German consumer’ would agree with the following statements?”	**Advantages**: + Generally a reduced sensitivity experienced by respondents when asked about other individuals + Useful when target individual is unable to self-report (e.g., due to illness, or disability) **Disadvantages**: - Potential for reduced data accuracy due to: • Bias from proxy’s personal beliefs or assumptions • Limited insight into internal states (e.g., emotions, attitudes) • Incorrectly labeling the proxy as more accurate • Tendency to portray proxies known to respondent more positively

Gergely and Rao (2021)*, Jang and Irwin (2020), Kilian and Mann (2021)*, Kotzur et al. (2020)*, Lehrer et al. (2019)*, Lopez-Becerra and Alcon (2021)*, Vassilopoulos et al. (2020)

*Significantly reduced SDB by proxy reporting

**Table 10:** Emphasizing honesty description, example operationalization, advantages, disadvantages and papers of publication

Description	Example operationalization	Advantages and disadvantages
Participants are explicitly asked to answer survey questions honestly or asked to pledge being honest. Following self-prophecy theory promising to be honest reduces dishonest answering (e.g., McDonald et al. 2017)	Example from McDonald et al. (2017, p.139) In a pre-election survey, participants were asked: Control condition: Are you going to eat dinner at home tomorrow? Experimental condition: “Are you willing to keep track of whether you voted in the upcoming November election and to be honest when you report this?” During a post-election survey, respondents were asked whether or not they voted in the November election.	**Advantages:** + Simple to implement with minimal changes to survey design + Cost-effective and unobtrusive **Disadvantages:** - May be overlooked in online survey formats - Limited empirical evidence - Potentially less effective in high-stakes contexts

Bir and Widmar (2020), McDonald et al. (2017)*, Vésteinsdóttir et al. (2019)*

*Significantly reduced SDB by emphasizing honesty

**Table 11:** Enhancing anonymity description, example operationalization, advantages, disadvantages and papers of publication

Description	Example operationalization	Advantages and disadvantages
In the enhanced anonymity approach, researchers typically manipulate the level of anonymity participants have when completing a survey. Anonymity can be varied by requiring participants to consent to: responding anonymously, responding confidentially, or responding confidentially with the collection of register data (Andersson et al. 2021). In laboratory settings, anonymity can also be manipulated by adjusting the degree of interaction with the researcher or the extent to which participants’ responses are visible to the researcher (Kogler et al. 2020). Providing more anonymous research environments is generally expected to reduce social desirability bias (SDB) compared to conditions with limited or no anonymity.	Example from Kogler et al. (2020, p. 393–394) Control condition: Participants interacted with the experimenter several times in person. In addition, doors of a cubicle in which the experimental task was carried out needed to stay open. The experimenter would also see the outcome of the experimental task, as participants were paid in person in the cubicle. Experimental condition: Participants did not interact with the experimenter and doors of the cubicle needed to stay closed during the experimental task. Individuals were informed that there was an experimenter present in the control room who could be contacted via an intercom system in case of any problems. They would not see this person. Full anonymity was ensured during payment by placing a sticker with a random number-letter code in each cubicle.	**Advantages**: + Perceived anonymity increased/emphasized + Easy implementation across different settings (e.g., online, lab) **Disadvantages**: - In online surveys, anonymity is often already guaranteed by researcher / assumed by participants - May not eliminate all forms of SDB, especially if participants still fear indirect identification - Can limit follow-up or data linkage if full anonymity is provided

Andersson et al. (2021), Eberlen et al. (2019), Kogler et al. (2020)

*Significantly reduced SDB by emphasizing honesty

**Table 12:** Time constraints description, example operationalization, advantages, disadvantages and papers of publication

Description	Example operationalization	Advantages and disadvantages
Time constraints are often manipulated by limiting the time participants have to answer a question. Some studies do not manipulate time directly but instead measure participants’ perceived time pressure (e.g., Brenner 2017). While it is generally assumed that socially desirable responses require less time (e.g., Protzko et al. 2019), this can vary depending on the task. For example, when participants are instructed to fake good (i.e., deliberately providing socially desirable responses) longer response times are typically observed (e.g., Roma et al. 2020)	Example of Roma et al. (2020, p. 252–253) Control condition: After reading each item, you should take all the time you need to respond most accurately, according to the instruction. Experimental condition: After reading each item, you should respond as quickly as possible. A short response time is important for this test. They explored whether speeded response time led to more socially desirable responses	**Advantages**: + Limiting response time may reduce deliberate impression management + Useful in controlled lab settings to explore cognitive mechanisms behind responding to survey questions **Disadvantages**: - May be impractical or unnatural in real-world or applied survey contexts

Brenner (2017)*, Protzko et al. (2019), Roma et al. (2020)

*Significantly reduced SDB by manipulating time constraints

**Table 13:** Interviewer influences description, example operationalization, advantages, disadvantages and papers of publication

Description	Example operationalization	Advantages and disadvantages
Interviewer characteristics or behavior can sometimes induce or reduce SDB. For instance, high levels of rapport experienced between interviewee and interviewer have been found to lead to higher rates of socially desirable responding (e.g., Horsfall et al. 2021). However, respondents familiar with interviewers have been found to respond less desirable to sensitive items (Kühne 2018).	Example from Horsfall et al. (p. 4–5). Experienced rapport was measured after a (face-to-face) interview, among interviewees as well as interviewers. The authors argued that a mutually pleasant experience reflected a high level of rapport between the interviewer and the respondent, given that the study itself focused on depression and anxiety (i.e., generally not considered to be pleasant topics to discuss). When a high level of rapport was experienced for both interviewee and interviewer, rapport was judged to be high. When a low level of rapport was experienced for one or both, rapport was judged to be low. Then, they investigated differences in socially desirable responses between the high-rapport group and the low-rapport group.	**Advantages**: **+** Using interviewers can lead to: • Higher response rates • Less satisficing • Ability to use more complex questionnaires **Disadvantages**: **-** Using interviewers can lead to: • Higher costs (e.g., compared to self-administered surveys) • Less standardized questionnaires, making results more difficult to compare • Increased risk of SDB

Horsfall et al. (2021)*, Kühne (2018)*, Leichtmann and Nitsch (2021)

*Significantly reduced SDB by manipulating interviewer characteristics of behavior

**Table 14:** Bogus pipeline procedure description, example operationalization, advantages, disadvantages and papers of publication

Description	Example operationalization	Advantages and disadvantages
In the bogus pipeline procedure, participants are informed that the researcher has access to a lie detecting procedure or device that can monitor the accuracy of participants’ responses—while in reality, the researcher has no objective measure for this. It is assumed that the feeling of getting caught in a lie is a larger cost to the participant than admitting the sensitive behavior, hence reducing SDB (e.g., Ward and King 2018).	Example of Ward and King (2018, p. 228) An experimenter taped sensors to the participants’ left cheek and inner wrist. Sensors were attached to a machine that lit up, appearing functional. Control condition Participants were instructed that the purpose of the sensors was to assess muscle activity and were shown fake feedback as an example of the machine’s functionality Experimental condition Participants were told that the sensors detected muscle movement associated with deception. They completed a “guilty knowledge test” ostensibly to calibrate the machine.	**Advantages**: + Can create a psychological pressure to be honest + Applicable across behavioral domains **Disadvantages**: - Ethical concerns due to use of deception (e.g., fake lie detectors) - Impractical for large-scale or field studies; requires controlled lab environment - Unnatural survey environment and limited real-world applicability

Jones and Elliot (2017), Sassenrath (2020)*, Ward and King (2018)

*Significantly reduced SDB by using bogus pipeline procedure.

**Table 15:** Survey sponsor description, example operationalization, advantages, disadvantages and papers of publication

Description	Example operationalization	Advantages and disadvantages
The survey sponsor/ organization can be manipulated in order to induce or reduce SDB. Information provided about the sponsor of the survey can change the social context in which participants answer questions, which in turn is expected to influence survey responses towards what they think the survey sponsor would prefer (e.g. Lüke and Grosche 2017)	Example from Lüke and Grosche 2017 (p.41) Attitudes towards inclusion (ATI) were measured for four experimental groups, varying in their survey sponsor. Below, two conditions are outlined. Condition A. Survey sponsor: ‘University of Potsdam – Institute of Inclusive Education (with no further comment about the ATI of the organization). Condition B. Survey sponsor: fictitious organization named ‘No Experiments with Our Children’. The statement referred to the threat of lowering the standards for all children in general education schools if pupils with special educational needs were placed in general classrooms (imminent risk of deteriorating standards in regular schools)	**Advantages**: + Simple to implement with minimal changes to survey design + Low-cost manipulation + Applicable across behavioral domains **Disadvantages**: - Context-dependent effects: the same sponsor may reduce bias in one group but increase it in another - While minimal deception, ethical concerns as participants are misled about the survey sponsor

Leeper and Thorson (2020), Lüke and Grosche (2017)*

* Significantly reduced SDB by using survey sponsor

**Table 16:** Vignette description, example operationalization, advantages, disadvantages and papers of publication

Description	Example operationalization	Advantages and disadvantages
Vignettes are hypothetical scenarios, characters or products that have to be evaluated on several, sometimes differently composed, characteristics. They enable researchers to ask questions about sensitive topics/attributes, in a more unobtrusive, indirect manner, through presenting hypothetical situations. As such, vignettes are assumed to reduce SDB (Walzenbach 2019)	Example from Walzenbach (2019, p. 105–106) Split-half design: the sensitive dimension, religion (Christian, Muslim, none), varied within or between subjects. Every vignette contained a description of a couple that used public services. They differed on several characteristics (in bold), including the sensitive dimension (in italics): “The child’s mother is working part time, the father is working full time. The parents and the child are living together in a household. This household’s overall monthly net income is 2800 Euro. The child’s grandparents are not available to help with childcare. The family has always lived in Konstanz and belongs to a Christian community. The fee for the day-care facility is 100 Euro per month.” Respondents were asked to evaluate on a Likert-scale how justified certain childcare fees were	**Advantages**: + Respondents may feel more comfortable evaluating hypothetical scenarios than answering direct questions + Flexible design: allows controlled manipulation of multiple variables within a single scenario + Applicable across behavioral domains **Disadvantages**: - Hypothetical nature may limit real-world validity - Complex design: requires careful design to ensure clear, realistic, and relevant scenarios - Participants may interpret vignettes in varied ways, introducing uncertainty in measurement accuracy

Horiuchi et al. (2019)*, Walzenbach (2019)

* Significantly reduced SDB by using vignette

**Table 17:** Subtle wording description, example operationalization, advantages, disadvantages and paper of publication

Description	Example operationalization	Advantages and disadvantages
Subtle wording refers to phrasing survey items in a way that obscures their true purpose, making them less obvious to respondents. This technique involves using indirect language to disguise sensitive or stigmatized topics in combination with filler items. The method is based on the assumption that participants are more likely to endorse covert or implicit items than overt, explicit ones, and as such, reduce social desirability bias (Thelan and Meadows 2022)	Example from Thelan and Meadows (2022, p. 19) This study compared scores on Rape Myth Acceptance Scales, the IRMA-2011 (older version of the scale) and IRMA-S (improved version of the scale) and related them to a social desirability scale in a within-subject design. Also, items were added that focused less on the sensitive topic itself (e.g., filler items). Example items of IRMA scales (italics added): IRMA-2011: 1. When guys rape, it is usually because of their strong desire for sex. 2. If a girl acts like a slut, eventually she is going to get into trouble. IRMA-S: 1. When men force women to have sex, it is usually because they cannot control their desire for sex. 2. If a woman sleeps around, eventually something bad is going to happen to her	**Advantages**: + Reduces item transparency, making the purpose of the sensitive item less obvious + Applicable across behavioral domains + Simple to implement in survey after item design **Disadvantages**: - Risk of interpretation ambiguity: covert or implicit phrasing may confuse respondents or lead to inconsistent understanding - Potential loss of precision: obscured items may not measure target constructs as directly or accurately

Thelan and Meadows (2022)

### Quality of the studies

#### Overall study quality

Quality assessments were carried out based on the criteria described in Table 3 (Sect.  2.6). Each of the ten study quality characteristics was scored with 0, 0.5 or 1, so the total score for each experiment was minimally 0 and the maximally 10. When a certain quality characteristic was *not applicable* and did not affect study quality (e.g., a random distribution of participants to conditions was not applicable for a within-subject design), we gave a score of 1. When the quality characteristic was *not reported* for the experiment or unclear, we gave a score of 0. Therefore, it should be noted that scoring 0 on some study quality characteristic was not always indicative of poor study quality in relation to experimental execution, but could also be indicative of less transparency with regard to reporting of important information. Overall, scores were high, with two-thirds of experiments scoring at least a 7.5 out of 10 and no experiment receiving less than 5 out of 10 points. The 3 experiments that scored a 5 manipulated the mode of administration. They all scored 0 on representativeness, random and even distribution of participants, participant description and mentioning of outliers. Of the 23 experiments scoring a 9.5 or higher, 15 were face-saving experiments (65%), 6 were list experiments (26%), 1 emphasized honesty (4%) and 1 used a probability-based technique (4%). See Table 18 for a full overview of the distribution of experiments in score frequencies.

**Table 18:** Overall distribution of score frequencies

Score	*N* (%)
0–4.5	0 (0)
5	3 (2)
5.5–6	7 (6)
6.5–7	29 (24)
7.5–8	29 (24)
8.5–9	30 (25)
9.5–10	23 (19)
Total	121 (100)

#### Study quality per characteristic and method

To get an overview of the study quality characteristics separately, we calculated the proportional score for each study quality characteristic separately (i.e., with a minimum of 0% and maximum of 100% score for each study quality characteristic). In addition, we calculated for each method a proportional score of each characteristic. Table 19 gives an overview of these proportional scores for each characteristic in total, and for each characteristic per method.

Again, the overall quality of the experiments was considered high, with particularly high quality assessments for six criteria: providing a clear description of the intervention, manipulations and measures, random distribution, the appropriateness of the statistical tests carried out, and having a clear description of the main findings, with scores ranging from 83 to 100%. The lowest quality assessments were found for representativeness of participants, and whether outliers were reported, with scores of 42% and 54%, respectively. Intermediate scores were found for a clear description of participant characteristics, and whether participants were evenly/properly distributed, with 71% and 68% of the maximum score.

The overall proportional score of the experiments was 81% (i.e., for all methods and all characteristics together). The best overall quality assessment was by far found for the face-saving method (i.e., 98% of the maximum score), followed by the list experiment (86%) and emphasizing honesty (85%). Overall, no method had a lower accumulated proportional score than 66% of the maximum score (i.e., mode of administration). Please note that not each quality criterion may be equally important in each type of experimental setting and/or method. Our aim was to provide a general quality assessment framework that was applicable across the diverse methodological approaches included in our review. Individual criteria can be weighted differently, as deemed relevant, using the numbers presented in Table 19.

**Table 19:** Proportional scores (0–100%) by evaluation characteristic and method

Study quality characteristic	Participant clearly described	Participant representative	Random distribution	Even or proper distribution	Description intervention	Manipulation	Measure	Statistical tests	Outlier	Main findings	Total
Overall	71	42	84	68	99	97	96	98	54	100	81
List	57	64	97	72	100	100	97	95	76	100	86
Probability	97	8	68	68	100	100	97	100	89	100	83
Face-saving	86	94	100	100	100	100	100	100	94	100	98
Mode	59	41	56	38	97	81	84	97	6	100	66
Proxy	85	23	77	38	96	92	96	100	15	100	72
Honesty	80	30	100	80	100	100	100	100	60	100	85
Anonymity	50	0	75	50	100	100	100	75	50	100	70
Time	25	25	100	50	100	100	100	100	0	100	70
Interviewer	67	71	100	67	100	100	83	100	0	100	73
Bogus	100	0	67	100	100	100	100	100	0	100	77
Sponsor	83	0	100	67	100	100	100	100	0	100	75
Vignette	0	33	100	100	100	100	100	100	33	100	77
Subtle	100	0	100	100	100	100	100	100	0	100	80

### Methods supported

Table 20 shows the frequencies and relative frequencies of the methods used for SDB reduction and the number of papers in which the experiments were reported. In addition, the table shows frequencies and relative frequencies of experimental results. These results are categorized as:

- *SDB-reduction supported *significant reduction of SDB in experimental condition as compared to control condition. This reduction took place for a majority or all items (i.e., the dependent variable).
- *SDB-reduction mixed/unclear *contradictory effects within a single experiment. There was no clear majority of items that found an effect, no effect or reversed effect of SDB reduction.
- *SDB-reduction unsupported *no significant reduction of SDB, with no effect or a reversed effect in experimental condition as compared to control condition. This absence of effect or reversed effect took place for a majority or all items.

The top five most frequently used methods, ordered from more to less frequent, were the list experiment, probability-based techniques, face-saving strategies, modes of administration and proxy reporting. List experiments were reported in 29 experiments in 20 different papers (about a quarter of the total number of papers and experiments). Approximately half of list experiments were effective in reducing SDB, a third found mixed or unclear results and around one fifth of the experiments did not find any support for the list method.

Probability-based techniques (RRTs/NRRTs) were carried out in 19 experiments and reported in 12 papers (making up around one-sixth of the experiments and number of papers). Looking at the number of experiments, this method was found effective approximately four out of ten times. It should be noted that one of the papers consisted of 8 experiments hypothesizing and finding the RRT would *increase* SDB based on their experimental design (John et al. 2018). Hence, while the method is a SDB reduction method, the experimental conditions were manipulated in such ways that the authors expected the method not to reduce SDB, but rather to increase SDB (i.e., less valid estimates of behavior). If we do not take into account these experiments, the effectiveness of the (non-)RRT rises to almost three-quarters of the experiments.

Face-saving strategies were reported in 18 experiments in 4 papers (around one-sixth of the experiments, and around one-twentieth of the papers). The average number of experiments (4.5) is highest for this method due to the fact that one of the papers reported 12 experiments. In all experiments, a significant effect was found of the method on reducing SDB. This makes the face-saving strategy the most successful method in reducing SDB compared to all other reported methods.

The mode of administration was investigated in 16 experiments reported in 16 papers (around one-eighth of the experiments, and one-fifth of the papers). Exactly half of the experiments found the hypothesized mode to be significant in SDB reduction. Most often, the difference in SDB between face-to-face interviews (more SDB expected) and self-administered online surveys was investigated (less SDB expected). One-third found mixed or unclear results and nearly one-fifth of the experiments did not find any differences. Proxy reporting took place in 13 experiments and 7 papers (somewhat more than one-tenth of the experiments, and close to one-tenth of papers). The method was successful in SDB reduction in somewhat less than half of the experiments.

**Table 20:** Frequencies and relative frequencies of methods for experiments and number of papers

Method	*N* total experiments (%)	*N* SDB-reduction supported (%)	*N* SDB-reduction mixed/unclear (%)	*N* SDB-reduction unsupported (%)
List	29 (24)	15 (52)	8 (28)	6 (21)
Probability	19 (16)	8 (42*)	1 (5)	10 (53)
Face-saving	18 (15)	18 (100)	–	–
Mode	16 (13)	8 (50)	5 (31)	3 (19)
Proxy	13 (11)	6 (46)	2 (15)	5 (38)
Honesty	5 (4)	4 (80)	–	1 (20)
Anonymity	4 (3)	–	–	4 (100)
Time	4 (2)	1 (25)	1 (25)	2 (50)
Interviewer	3 (3)	2 (50)	1 (25)	–
Bogus	3 (2)	1 (33)	2 (67)	–
Sponsor	3 (2)	2 (67)		1 (33)
Vignette	3 (2)	2 (67)	1 (33)	–
Subtle	1 (1)	–	1 (100)	–
Total (%)	121 (100)	67 (55)	22 (18)	32 (26)

*Including John et al.’s (2018) experiments aimed at inducing SDB. Excluding experiments of John et al. (2018) would lead to an increase of relative effectiveness of the method to 73% of all experiments

### Topics

In total, at least 17 behavioral and/or cognitive topics that could be grouped together were investigated in the 121 experiments (i.e., there were 8 experiments looking into multiple topics). Table 21 shows all topics investigated and frequencies of method supported, mixed/unclear or unsupported for each topic. The topics most often investigated were health, stereotypes, politics, sustainability and illegal behavior. Together, these five topics made up more than two-thirds of the experiments (70%). Experiments looking into the topics investigated more than twice that had a success rate in reducing SDB in 50% or more were health, politics, sustainability and multiple topics. Experiments looking into the topics investigated more than twice that had a success rate in reducing SDB of less than 50% were stereotypes, cheating, prosocial behavior and personality measures.

**Table 21:** topics investigated and frequencies of method supported, mixed/unclear or unsupported per topic

Topic	Total (%)	SDB-reduction supported (%)	SDB-reduction Mixed/unclear (%)	SDB-reduction unsupported (%)
Health	28 (24)	21 (75)	3 (11)	4 (14)
Stereotypes	25 (20)	12 (46)	12 (50)	1 (4)
Politics	15 (12)	10 (67)	–	5 (33)
Sustainability	9 (7)	6 (67)	1 (11)	2 (22)
Illegal behavior	8 (7)	4 (50)	1 (13)	3 (38)
Multiple topics	8 (7)	5 (63)	–	3 (38)
Cheating (e.g., games, tests)	6 (5)	1 (17)	–	5 (83)
Prosocial behavior	5 (4)	1 (20)	–	4 (80)
Personality measures	5 (4)	–	1 (20)	4 (80)
Religion	2 (2)	–	2 (100)	–
Law enforcement	2 (2)	2 (100)	–	–
Education	2 (2)	2 (100)	–	–
Animal welfare	1 (1)	1(100)	–	–
Relationship behavior	1 (1)	–	–	1(100)
Athletic behavior	1 (1)	1(100)	–	
Human-robot interaction	1 (1)	–	1(100)	–
Sexual behavior	1 (1)	–	1(100)	–
Ethical behavior	1 (1)	1 (100)	–	–
Total	121 (100)	67 (55)	22 (18)	32 (26)

## Discussion

The objective of this study was to evaluate the effectiveness of Social Desirability Bias (SDB) reduction methods through a systematic review of the literature. We found a total of 121 experiments aimed at reducing SDB published in 79 peer-reviewed papers for the period 2017 up to and including 2021. These experiments were conducted in more than 20 Western countries, and addressed over 17 distinct behavioral or cognitive domains. Quality of experiments was generally high, based on 10 quality measures regarding participants, manipulations, measures, statistical analysis and the description of findings. Our findings underscored variability in the utilization and effectiveness of SDB-reduction methods, both across and within methods. The most prevalent approaches included list experiments, probability-based methodologies, face-saving strategies, mode of administration, and proxy reporting, constituting 79% of all included experiments.

### Face-saving strategies

By far the most effective SDB reduction method appeared to be the face-saving strategy, seemingly the method of choice when one aims to reduce SDB. All 18 experiments included in this review that used this method found a significant SDB reduction. A specific advantage of the face-saving strategy is that writing a face-saving preamble/using forgiving wording for survey questions is achievable for a broad spectrum of behavioral topics, rendering it applicable to numerous, if not all, behavioral topics one can think of. Hence, the method can be easily applied to survey research looking into other behavioral topics. One possible disadvantage is that formulating face-saving answer options can be quite complex. For instance, the guilt-free answer options “only when necessary” and “occasionally” (i.e., as used by Daoust et al. 2021a, b) are not suitable for all types of questions on all kinds of topics. Firstly, questions for which an agreeing response is socially desirable cannot use guilt-free answer options like “only when necessary” and “occasionally”. Imagine we ask “Did you eat two pieces of fruit a day in the past 7 days?”, one has to design disagreeing guilt-free answer options (e.g., “no, but I tried to”/ “no, but I ate at least one piece a day”). Secondly, when one asks about topics where social norms and perceptions fluctuate, there is an inherent complexity in determining what constitutes socially desirable and undesirable responses. While this is a more general issue across methods, it can make formulating and interpreting guilt-free answer options specifically challenging. If we take another example: “How often do you test for a STD?”, selecting answer options like ‘only when necessary’ or ‘occasionally’ could project socially desirable behavior instead of socially undesirable behavior, as one could argue regular STD testing aligns with health-conscious practices and societal expectations. On the other hand, paradoxically, within other contexts, disclosing such behavior might be perceived as socially undesirable (as one implicitly admits that one behaved in such a manner that it is necessary to test for a STD) (cf.: King 2022). Therefore, it is recommended to investigate social norms in the population of your interest closely, before formulating guilt-free answer options, especially for such context-dependent topics.

### List experiment

Other promising methods were the list experiment and probability-based techniques. The list experiment was found to be effective in half of the experiments (52%). With a quarter of all experiments/papers included in this review, it was the most often employed method.

With approximately half of the experiments effectively reducing SDB, the method does not appear as effective as the face-saving strategy. Still, a recent meta-analysis (Li and Van den Noortgate 2022) which synthesized 246 effect sizes underscores the comparative efficacy of the list experiment against direct questioning. In this review, list experiments were applied in a more diverse set of behavioral domains compared to face-saving, being conducted in the field of stereotypes and politics (e.g., Thomas et al. 2017; Kleykamp et al. 2018; Mueller 2021) and in the fields of health, sustainability and illegal behavior (e.g., Munzert and Selb 2020; Beiser-Mcgrath and Bernauer 2021; Agerberg 2020).

The assumed greatest advantage of the list experiment is the level of anonymity provided, as respondents are not required to disclose their agreement with the sensitive item specifically.

To uphold this anonymity, it is crucial that the four non-sensitive items within the list exhibit a balanced design (i.e., half of the items should be likely to obtain agreement from participants, while the remaining half should not). This approach mitigates the risk of either complete agreement or disagreement with the items, which would undermine the assumption of anonymity (i.e., complete agreement or disagreement reveals (dis)agreement on the sensitive statement as well).

List experiments also have disadvantages. For instance, one needs a large sample size for reaching sufficient statistical power and obtaining precise estimates. Moreover, lists can suffer from design effects. A design effect occurs when respondents evaluate a statement (i.e., list item) relative to another, making (dis)agreement on a previous statement influence their (dis)agreement on one of the next list statements. In addition, the “no liar” assumption of list experiments, the assumption that respondents will answer truthfully to the control items, can be disrupted by participant’s deliberate misreporting. Moreover, list experiments have the disadvantage of only uncovering agreement for the sensitive item on an aggregated level (e.g., one does not know agreement with the sensitive statement for a specific participant). Lastly, analyzing list experiments can be quite comprehensive (e.g., modeling of both sensitive and control items, design effects, ceiling and floor effects). Attempts have been made to address these drawbacks (e.g., see Glynn 2013; Blair and Imai 2012; Chou et al. 2020; Blair et al. 2020). Scholars and practitioners intending to conduct list experiments are advised to carefully deliberate on experimental design and formulation of list items. They are directed to consult Glynn (2013), Blair and Imai (2012), Chou et al. (2020) and Blair et al. (2020) for guidance on best practices in this regard.

### Probability-based techniques

Probability-based techniques, which include randomized and non-randomized response techniques (RRTs & NRRTs), comprised approximately 15% of all included experiments.

In general, (N)RRTs were relatively dispersed over behavioral topics, but most often carried out in the context of cheating (e.g., solving mathematical problems, trivia questions), followed by illegal behavior, health and stereotypes. Just as for the list experiment, probability-based techniques have the advantage of a greater perceived sense of anonymity, as the participant does not directly answer the sensitive statement (Blair and Imai 2012). Of all experiments using a probability technique, 42% showed significant SDB reduction.

Of the 11 RRT experiments included in this review, 8 specifically focused on testing conditions under which the RRT was expected to fail. These 8 experiments were part of a study of John et al. (2018), who investigated circumstances in which RRTs yield less accurate and less precise estimates compared to direct questioning. A key finding was that participants’ concerns about their answers being misinterpreted led to unreliable results. Specifically, when participants deliberately disregarded the randomization procedure, reliable estimates could not be calculated. Non-adherence was especially common when the randomization required participants to appear as though they were admitting to socially undesirable behavior, even if their true behavior was socially desirable. In such cases, participants feared their responses would be misread as indicating engagement in a behavior they had not actually committed. John et al. (2018) suggested a modification to address this issue, proposing an adjustment in response labels to enhance data quality. For instance, repeating parts of the randomization question in addition to standard yes/no answer options. For detailed information, readers are directed to John et al. (2018). Among the remaining three RRT experiments included in this review, only one explicitly recommended the use of RRT over direct questioning.

In the case of the 9 NRRT experiments, 6 reported a significant reduction in SDB. However, it is important to note that only two of these experiments compared estimates to a known true value of the behavior. These experiments measured cheating in a task (e.g., anagram solving; Meister et al. 2020a), which has limited ecological validity. The remaining NRRTs validated their findings using a control attribute and/or the more-is-better assumption. For example, if the estimated prevalence of a nonsensitive control question (e.g., “I was born in November or December”) matched its known prevalence, this was interpreted as evidence that participants understood and followed the question format correctly (e.g., Hoffmann and Jochen 2019). In addition, the “more-is-better” assumption holds that higher admission rates of socially undesirable behavior in an alternative format (i.e., the experimental condition) compared to a direct question format (i.e., control condition) indicate superior validity. While authors commonly use and try to justify this assumption, it is not universally reliable—particularly for indirect techniques such as list experiments and (N)RRTs (e.g., Höglinger and Jann 2018; Jerke et al. 2019). In sum, based on the RRT and NRRT experiments included in this review, no firm conclusions can yet be drawn about their overall effectiveness.

RRTs and NRRTs share several disadvantages with list experiments: they require large sample sizes, and estimates of socially desirable responding (SDB) are based on aggregated data—meaning that no individual-level information is available on the sensitive item. Moreover, (N)RRT and list designs suffer from a bias-variance trade-off (Warner 1965). That is, while direct questions may exhibit bias, they typically have lower variance. Conversely, alternative question formats such as lists and (N)RRTS might introduce less bias but are associated with higher variance. As an example, list designs generate approximately 14 times more noise compared to direct questions (Blair et al. 2020). This trade-off implies that a large sample size and/or anticipated bias is necessary for justifying the use of list or (N)RRT methods.

In the case of RRTs specifically, prior research has demonstrated that while they can reduce socially desirable responding (SDB), their use is also accompanied by substantial limitations. Prevalence estimates often display large variances and are difficult to compare—even when based on similar items and samples (Lensvelt-Mulders et al. 2005). Moreover, issues such as non-compliance, refusals to answer, and misunderstandings of the instructions have been widely documented, leading to implausible or distorted estimates (e.g., Coutts and Jann 2011; Edgell et al. 1982; Kirchner 2015; Krumpal 2012; Ostapczuk et al. 2011; van der Heijden et al. 2000; John et al. 2018).

While NRRTs alleviate some concerns of the RRT’s by reducing cognitive burden (i.e., by integrating the randomization directly into the answer options) they are also not without limitations. For instance, the most commonly used NRRT design does not allow researchers to clearly distinguish between genuine reductions in SDB and alternative explanations such as random responding or non-compliance, making validation problematic (Walzenbach and Hinz 2019, 2023). False positives can be found for NRRTs as well (e.g., Höglinger and Jann 2018; Wu and Tang 2016). There is also some evidence that higher educated samples adhere to instructions better (e.g., Meisters et al. 2020a), suggesting the method may be less suitable for lower-educated samples. In our review, however, the evidence was inconclusive: based on an examination of the five student samples examined included in our review, three showed reductions in SDB, while two did not.

### Mode of administration

Manipulating mode of administration proved effective in reducing SDB in around half of the experiments. It should be noted that while these experiments had the lowest quality score on random assignment of respondents to conditions (56%), many studies without randomization implemented statistical adjustments—such as weighting, matching, or covariate balance checks—to facilitate valid comparisons across survey modes (e.g., Knox et al. 2020; Abrajano and Alvarez 2019; Zhang et al. 2017; Liu 2017). These non-randomized studies typically involved comparisons across distinct sampling frames rather than allowing respondents to self-select into modes. Accordingly, the lower score on random assignment should be interpreted in light of the corrective measures most of these studies employed.

The majority of mode experiments looked into a comparison of self-administered modes and face-to-face interviews and found a significant advantage for self-administered surveys (e.g., Berzelak and Vehovar 2018; Zhang et al. 2017; Abrajano and Alvarez 2019; Klein et al. 2020; Knox et al. 2020; Cea D’ancona). However, it is important to note that there are also advantages in face-to-face interviews, such as higher response rates and enhanced accessibility of hard-to-reach populations (Haan and Ongena 2014).

In addition, while online survey administration may help reduce SDB, it also presents several methodological challenges. First, online surveys are known for exhibiting higher non-response rates compared to other modes of administration, which can lead to missing data and reduced representativeness (Fan and Yan 2010; Bethlehem 2010). However, declining response rates are not exclusive to online surveys and recent research suggests that non-response trends over the past two decades in European surveys have even been comparable across multiple modes (Jabkowski and Cichocki 2025). A challenge more specific to online formats is the limited control over the respondent’s environment: participants may complete surveys in distracting settings or while multitasking, potentially compromising data quality (Zwarun and Hall 2014; Wright 2005). Additionally, online surveys that are long are vulnerable to respondent fatigue, where participants lose focus or motivation over time, increasing the likelihood of careless or incomplete responses (Ghafourifard 2024; Jeong et al. 2022). Online surveys are also susceptible to satisficing: a behavior in which respondents choose acceptable but suboptimal answers to reduce cognitive effort instead of thoughtfully engaging with each question (Krosnick 1991). Nonetheless, recent evidence from a cross-national study found no particular increase in satisficing behavior in online surveys compared to other modes across four countries (Clement et al. 2023). In summary, online surveys can have potential risks and there can be trade-offs with other modes. Researchers and practitioners must carefully consider these before choosing a mode of administration.

### Proxy experiments

With regard to proxy experiments, the operationalization of proxy measures exhibited considerable variation across studies, with no specific operationalization type that could be identified as leading most often to significant SDB reduction. Therefore, attempting to uniformly summarize proxy reporting as a method or provide recommendations on operationalizations for reducing SDB does not appear viable.

It is important to note that research into proxy reporting also found potential adverse effects of the method. Proxies can compromise data accuracy and reliability in multiple ways. A key issue is the frequent mismatch between proxy and self-reports due to the proxy’s personal beliefs or self-schemas, which can unconsciously bias their perceptions and responses. Additionally, proxies often struggle to accurately report on internal states—such as emotions, attitudes, or subjective experiences—because these are not directly observable. In addition, while proxies can be used to reduce SDB, they may also introduce it, particularly when participants have to report about people they know (i.e., they may portray this known individual in an overly favorable light). For reviews, see Baidoo et al. (2024) and Dagne et al. (2025).

### Other methods

There were methods that were carried out less frequently, yet seemed quite effective in reducing SDB (≥ 67%, emphasizing honesty, interviewer influences, survey sponsor manipulation and vignette usage as well as less frequently used methods that seemed not so effective (≤ 33%, the bogus pipeline procedure, time constraints, enhancing anonymity and subtle wording). While these methods were successful or unsuccessful based on relative numbers, absolute numbers are too small to draw any meaningful conclusions.

### Future directions and limitations

Further investigation can enhance future decision-making processes with regard for SDB reduction methods and contribute to the advancement of our theoretical understanding of the phenomenon. First, we recommend investigating the effectiveness of face-saving strategies further. While the method looks promising as an effective measure to reduce SDB, face-saving is tested only across a limited amount of behavioral topics in this review (*politics*: Setzler 2018; *stereotypes*: Charles and Datallo 2018; and *health*: Daoust et al. 2021a, b). Within these topics, diversity was also limited. The 15 face-saving experiments looking into health behavior all focused on adherence to COVID-19 restrictions, using practically identical operationalizations (Daoust et al. 2021a, b). Expanding the investigation of the potential effectiveness of face-saving strategies could consist of replication studies, including a wider range of topics and using different operationalizations. In addition, all studies included in this review looked into the effects of using a face-saving preamble exclusively, or in conjunction with guilt-free (agreeing) answer options. Studies investigating the separate effects of a face-saving preamble and offering guilt-free answer options could offer valuable insights into the distinct impacts of the latter.

In addition, future research into other methods included in this review could benefit from investigating the specific conditions under which they are more effective in reducing SDB, considering that all other methods have not consistently proven effective. In the case of list and (N)RRT designs, reducing measurement error could improve their performance. For instance, for lists, one could further explore optimizing list lengths and item contents (i.e., involving experimentation with lengths and items to find an optimal balance between bias reduction and variance minimization). For RRTs, error may be also reduced by improving procedures and survey formulations, in line with recommendations by John et al. (2018) and meta-analytic findings like Lensveldt-Mulders et al. (2005). Enhancing explanation of randomization procedures can reduce cognitive burden and bolster participant trust (e.g., Jerke et al. 2019). For NRRTs, while there is considerable research activity into their effectiveness, and many variations exist (e.g., the crosswise model, triangular model, parallel model), determining which one works best remains somewhat unclear (Erdmann 2019; Wu and Tang 2016). More extensively testing the effectiveness of different NRRTs is therefore a relevant departure point for future studies.

Regarding proxy reporting, it is important to explore how to formulate more standardized operationalizations and determining the appropriate social referents (i.e., proxies) to utilize in different contexts of SDB reduction. For methods that were underexplored in this review, yet showed promise in terms of relative effectiveness, it is advised to extend experimental survey research, particularly for those that may be relatively straightforward to implement for practitioners (e.g., methods like emphasizing honesty and manipulation of survey sponsors).

A further consideration, is that for all behavioral and cognitive topics investigated, there were only a few that were extensively studied. Only health, stereotypes and politics were each studied in more than 10 experiments (for more detailed information on topics, see supplementa material). Other societally relevant topics like sustainability, illegal behavior, prosocial behavior, animal welfare, sexual behavior and ethical behavior were only rarely explored. Future studies aiming to reduce SDB would benefit from a strategic shift towards investigating societally relevant topics that have received limited scrutiny thus far. Investigating underexplored topics with methods recommended based on this review could yield valuable insights. Furthermore, we did not systematically assess whether studies reported formal balance checks across experimental groups. Although many studies accounted for potential imbalances by including variables such as gender and age as covariates, the explicit reporting of balance checks would provide a stronger indicator of internal validity. On a more general note, in order to more critically assess the more-is-better assumption, more validation studies should be conducted among different methods and designs.

Lastly, it merits emphasis that for the sake of replicability of the search strategy we decided not to include any gray literature (i.e., unpublished work). As such, the current systematic review might suffer from publication bias, journal editors’ tendency to publish mostly positive results (i.e., finding significant results in the direction of the hypothesis; Thornton and Lee 2000). This could mean that the methodological quality of SDB reduction strategies, together with relative success in actually reducing SDB, may be exaggerated here. Nonetheless, there are indications to the contrary. Approximately half of the experiments (55%) in this review reported significant results, while the rest yielded mixed/unclear (18%) or non-significant outcomes (26%). Notably, 16 of the 19 multi-experiment papers included at least one experiment with no or mixed effects, and only 3 found full support across all experiments, indicating that publication bias does not fully preclude the reporting of null or mixed results.

### Conclusion

Based on this review we can make recommendations of future utilization of SDB reduction methods. First, we suggest incorporating face-saving strategies in survey research, especially when one looks into sensitive topics for which clear social norms exist and when yes/no questions can be formulated. Second, if a face-saving strategy is not deemed suitable for any reason (e.g., when it is difficult to formulate clear or fitting guilt-free answer options or preambles), yet one is able to formulate a question with dichotomous answer options (i.e., yes/no or true/false) one is advised to use list or NRRT methods. We recommend specifically a NRRT over a RRT as NRRTs come with less potential noise (i.e., interference that can affect the accuracy of the collected data, like participants’ deliberate non-adherence to instructions). In addition, NRRTs are generally easier to comprehend for participants than RRTs. If one does want to carry out a RRT, the reader is recommended to inspect John et al. (2018) for potential pitfalls and solutions. For (N)RRTs and list techniques one has to take into account that the design and/or analysis can be quite complex, and knowledge to set up and analyze such designs is required. Proxy reporting is a method that can be considered for topics with less clear social norms (for items with more than two answers, or when one does not have the time or resources to properly use one of the former methods). One has to take into account that there is still much uncertainty on the effectiveness of this method, as proxy items are not uniformly operationalized, and it seems to work only in close to half of the applications based on this review. Lastly, an overall recommendation is to use (online) self-administered surveys whenever possible, or, as an alternative, mixed-mode designs including a self-administered (online) mode). It is evident that each technique for reducing SDB in survey research possesses its strengths and weaknesses. As such, a careful consideration prior to method selection is necessary. Moreover, further investigation of these methods is warranted to facilitate informed decision-making.

In sum, this systematic review: demonstrates that certain SDB reduction methods, particularly face-saving strategies, can significantly enhance data accuracy in surveys involving sensitive questions; emphasizes the importance of carefully selecting SDB reduction techniques, tailored to one’s time and resources available, and; offers a foundation for refining and expanding SDB reduction methods across a broader range of topics, which is crucial for further enhancing the validity of self-report data in future research.

## Electronic Supplementary Material

Below is the link to the electronic supplementary material.

Supplementary Material 1

## Data Availability

The data that support the findings of this study are openly available in DANS (Data Archiving and Networked Services) at 10.17026/SS/G8JC8E.
