# Supplementary material for: Unraveling honest responding: a systematic review on the effectiveness of social desirability bias reduction methods in survey research
Source: Qual Quant. 2026 Mar 14;60(3):10359–91. doi: 10.1007/s11135-026-02664-7 (PMC13230293; doi:10.1007/s11135-026-02664-7)
Supplement: Supplementary file 1 — Supplementary Material 1 [file 11135_2026_2664_MOESM1_ESM.docx]

## Supplemental material

**Article title**: Unraveling Honest Responding: a Systematic Review on the Effectiveness of Social Desirability Bias Reduction Methods in Survey Research.

**Journal name**: Quality & Quantity

**Author names**

Emma Zaal^1^, Yfke Ongena^2^, Nina van der Velden^3^, Dan Loughnan^4^, John Hoeks^5^

^1,2,3,5^Faculty of Arts, University of Groningen, the Netherlands

^4^Faculty of Social Sciences, Radboud University, the Netherlands

**Corresponding author**: Emma Zaal, e.l.zaal@rug.nl

## Sample

Table S1 shows the distribution of countries in which the experiments were carried out (i.e., for both Austria and Spain, 5 experiments were carried out, together making up 10 experiments and 8.3% of the total sample).

Table S1: Country of experiment carried out

| Country | N (%) |
| --- | --- |
| U.S. | 52 (43.0) |
| Germany | 24 (19.8) |
| Austria; Spain | 10 (8.3) |
| Canada; the Netherlands | 8 (6.6) |
| Australia; Iceland | 6 (5) |
| Ireland; Poland; Sweden; U.K. | 8 (6.6) |
| Greece; Slovenia; Romania; Belgium; Cyprus; Denmark; Great Britain; France; Brazil*; Italy; New Zealand; E.U. Residents; Unknown. | 13 (10.7) |
| Total | 121 (100) |
| *We incorporated outcomes of one non-Western country, as it was part of a study with 12 experiments, 11 of which conducted in Western countries (Daoust et al., 2021b). | |

Table S2 shows that when a probability sample was used, two-thirds of the experiments found a significant reduction of SDB in the experimental condition. For non-probability samples, this was just over half of the experiments. In experiments in which a probability sample was employed, only 9% of the methods were unsupported (N=2), compared to 29% of the non-probability samples (N=30).

Table S2: Types of samples and effectiveness in reducing SDB.

| Type of sample | N total | N supported | N mixed/unclear | N unsupported |
| --- | --- | --- | --- | --- |
| Probability total | 21* (100%) | 14 (67%) | 5 (24%) | 2 (9%) |
| Cluster/Stratified | 16 | 10 | 4 | 2 |
| Random Digit Dialing | 3 | 3 |  |  |
| Unspecified | 2 | 1 | 1 |  |
| Non-probability total | 103* (100%) | 56 (54%) | 16 (16%) | 30 (29%) |
| Convenience | 52 | 23 | 9 | 20 |
| Quota | 38 | 27 | 3 | 8 |
| Purposive | 4 | 2 | 1 | 1 |
| Snowball | 1 | 1 |  |  |
| Unspecified | 8 | 3 | 4 | 1 |
| *NTotal=124, as three experiments used two sampling methods (e.g., convenience + snowball). | | | | |

Table S3 shows that samples below 250 participants (N=21, 17%) were least effective in reducing SDB, with a success rate of 24%. Samples between 250-499 and 500-999 participants were successful in reducing SDB in 62% of the experiments and 48% respectively. Samples larger than 1000 participants were successful in reducing SDB in at least two-thirds of the experiments (66.7%) and at most three-quarters of the experiments (75%).

Table S3: Sample sizes and effectiveness in reducing SDB

| Sample size | N total | N supported (%) | N mixed/unclear (%) | N unsupported (%) |
| --- | --- | --- | --- | --- |
| 0-249 | 21 | 5 (24) | 7 (33) | 9 (43) |
| 250-499 | 13 | 8 (61.5) | 2 (15.4) | 3 (23.1) |
| 500-999 | 25 | 12 (48.0) | 3 (12) | 10 (40) |
| 1000-1999 | 33 | 22 (66.7) | 5 (15.1) | 6 (18.2) |
| 2000-3499 | 12 | 9 (75) | 1 (8.3) | 2(16.7) |
| >3500 | 13 | 9 (69.2) | 2 (15.4) | 2 (15.4) |
| ? | 4 | 2 (50) | 2 (50) |  |

Table S4 shows that In 66 (55%) of the 121 experiments incentives were provided for participants, while there were 16 experiments (13%) in which no incentives were given. In 12 experiments (10%), only a part of participants was compensated for participation and for 27 experiments (22%) no information was provided on whether incentives were given or not. 75% of the experiments in which no incentives were given were successful in reducing SDB, while only 50% of the experiments in which incentives were given were successful. The lowest success rate in reducing SDB was found when only a part of the participants received incentives (42%).

S4: Incentives given and effectiveness in reducing SDB

| Incentives given | N total (%) | N supported (%) | N mixed/unclear (%) | N unsupported (%) |
| --- | --- | --- | --- | --- |
| No | 16 (13) | 12 (75) | 1 (6) | 3 (18) |
| Yes | 66 (55) | 33 (50) | 13 (20) | 20 (30) |
| Mixed | 12 (10) | 5 (42) | 4 (33) | 3 (25) |
| Unknown | 27 (22) | 1 (63) | 4 (15) | 6 (22) |
|  |  |  |  |  |

## Underreporting/Overreporting

Table S5 shows the distribution with regard to the underreporting of socially undesirable behavior and the overreporting of socially desirable behavior. Somewhat less than half of the experiments investigated topics in which underreporting was the socially desirable response (i.e., behaviors violating COVID-19 restrictions), somewhat less than a third of the experiments looked into overreporting (i.e., behaviors in compliance with COVID-19 restrictions), and somewhat less than a third of the experiments looked into both under-, and overreporting of behavioral and/or cognitive topics. In around two-thirds of the experiments investigating underreporting, the method that was used to reduce SDB was successful, against somewhat less than half of the methods used for overreporting and both underreporting & overreporting.

Table S5: Types of reporting and effectiveness in reducing SDB.

| Socially Desirable reporting = | Total (%) | N supported (%) | mixed/unclear (%) | N unsupported (%) |
| --- | --- | --- | --- | --- |
| Underreporting | 54 (45) | 36 (66.7) | 8 (14.8) | 10 (18.5) |
| Overreporting | 32 (26) | 15 (46.9) | 8 (25) | 9 (28.1) |
| Underreporting + Overreporting | 35 (29) | 16 (45.8) | 6 (17.1) | 13 (37.1) |

## Topics

Table S6.1 to table S6.5 shows for the 5 most often investigated topics which methods were employed, including the effectiveness of these methods in reducing SDB per topic.

In 28 health experiments, 6 methods were used for SDB reduction, of which three-quarters were found effective (table S6.1). The face-saving method was the only method that was always effective in reducing SDB when it came to health-related topics. Two-thirds of the probability-based techniques and three-fifths of mode of administration manipulations reduced SDB in health experiments. List experiments looking into health were less effective in reducing SDB, with a one-third success rate. Emphasizing honesty and enhancing anonymity were not found effective, yet both carried out only once for this topic.

S6.1: Method usage and effectiveness in reducing SDB for survey items on health

| Health | N total (%) | N supported (%) | N mixed/unclear (%) | N unsupported (%) |
| --- | --- | --- | --- | --- |
| Face saving | 15 (53.6) | 15 (100) |  |  |
| Mode | 5 (17.9) | 3 (60) | 2 (40) |  |
| List | 3 (10.7) | 1 (33.3) | 1 (33.3) | 1 (33.3) |
| Probability | 3 (10.7) | 2 (66.7) |  | 1 (33.3) |
| Anonymity | 1 (3.6) |  |  | 1 (100) |
| Honesty | 1 (3.6) |  |  | 1 (100) |

Within the 25 experiments on stereotypes that were examined, 5 different methods were used (table S6.2). Around half of these experiments were successful in reducing SDB. The most often used methods for this topic were the list experiment and mode manipulations, successful in somewhat less than half for the first, and exactly half for the latter method.

S6.2: Method usage and effectiveness in reducing SDB for experiments on stereotypes

| Stereotypes | N total (%) | N supported (%) | N mixed/unclear (%) | N unsupported (%) |
| --- | --- | --- | --- | --- |
| List | 11 (44) | 5 (45.5) | 6 (54.5) |  |
| Mode | 6 (24) | 3 (50.0) | 2 (40) | 1 (10) |
| Probability | 3 (12) | 2 (66.7) | 1 (33.3) |  |
| Proxy | 3 (12) | 2 (66.7) | 1 (33.3) |  |
| Face-saving | 1 (4) | 1 (100) |  |  |
| Vignette | 1 (4) |  | 1 |  |

There were 15 experiments looking into topics related to politics, using 7 different methods (table S6.3). Two-thirds of these 15 experiments actually reduced SDB. The list experiment was employed most often, and effective in reducing SDB for around two-fifths of the experiments. All other methods were fully successful in reducing SDB - yet, only carried out once or twice with regard to topics related to politics.

S6.3: Method usage and effectiveness in reducing SDB for experiments on politics

| Politics | N total (%) | N supported (%) | N mixed/unclear (%) | N unsupported (%) |
| --- | --- | --- | --- | --- |
| List | 8 (53.3) | 3 (37.5) |  | 5 (62.5) |
| Face-saving | 2 (13.3) | 2 (100) |  |  |
| Probability | 1 (6.7) | 1 (100) |  |  |
| Proxy | 1 (6.7) | 1 (100) |  |  |
| Vignette | 1 (6.7) | 1 (100) |  |  |
| Mode | 1 (6.7) | 1 (100) |  |  |
| Honesty | 1 (6.7) | 1 (100) |  |  |

Topics related to sustainable and unsustainable behavior were investigated in 9 experiments with 4 methods (table S6.4). Two-thirds of these experiments reduced SDB. Proxy reporting was used most often, and effective in 3 out of 4 experiments. List experiments and using vignettes were successful in reducing SDB in all experiments looking into sustainability - yet, these were only carried out twice and once respectively. Manipulating mode of administration was not found effective in two experiments for this topic.

S6.4: Method usage and effectiveness in reducing SDB for experiments on sustainability

| Sustainability | N total (%) | N supported (%) | N mixed/unclear (%) | N unsupported (%) |
| --- | --- | --- | --- | --- |
| Proxy | 4 (44.4) | 3 (75) |  | 1 (25) |
| Mode | 2 (22.2) |  | 1 (50) | 1 (50) |
| List | 2 (22.2) | 2 (100) |  |  |
| Vignette | 1 (11.1) | 1(100) |  |  |

The fifth most often investigated topic, illegal behavior, consisted of 8 experiments using 6 methods (Table S6.5). Half of the experiments were successful in reducing SDB. Methods that were successful in reducing SDB in the context of illegal behavior, all employed once, were asking for honesty, list experiments and proxy reporting. Probability-based techniques were used most often with regard to illegal behavior with a success rate of one-third in SDB reduction. Enhancing anonymity was not found successful in reducing SDB for this topic.

S6.5: Method usage and effectiveness in reducing SDB for experiments on illegal behavior

|  | N total (%) | N supported($) | N mixed/unclear (%) | N unsupported (%) |
| --- | --- | --- | --- | --- |
| Probability | 3 (37.5) | 1 (33.3) |  | 2 (66.7) |
| Anonymity | 1 (12.5) |  |  | 1 (100) |
| Honesty | 1 (12.5) | 1 (100) |  |  |
| List | 1 (12.5) | 1 (100) |  |  |
| Proxy | 1 (12.5) | 1 (100) |  |  |
| Subtle | 1 (12.5) |  | 1 (100) |  |
